# Retraction and replacement of: Strategic pathways to International Classification of Diseases, 11th Revision adoption in France and the United States

**DOI:** 10.1093/haschl/qxaf098

**Published:** 2025-07-01

This is a retraction and replacement of:

Bastien Boussat, Robert Jakob, Laurent Boyer, Patrick S Romano, Strategic pathways to International Classification of Diseases, 11th Revision adoption in France and the United States, *Health Affairs Scholar*, Volume 3, Issue 3, March 2025, qxaf037, https://doi.org/10.1093/haschl/qxaf037

Shortly after article publication, the Authors of this article alerted the Editorial Office to the fact that the ICD-11 Workgroup under the National Committee on Vital and Health Statistics was suspended on or around January 16, 2025, in anticipation of President Trump's notice of the United States’ intended withdrawal from the World Health Organization (WHO). As a result, one key citation is no longer available, the content of the Workgroup's discussions related to payment programs should be corrected, and the past tense is more appropriate than the present perfect tense in describing the Workgroup's activities. The specific changes are outlined below.

- On page 2, “has focused” was changed to “focused” in the following sentence: “The ICD-11 Workgroup process focused on information-gathering by reviewing research studies and findings, soliciting input from subject matter experts, and engaging all key stakeholders.”
  - The following citation was also added as reference 10: NCVHS ICD-11 Workgroup on Timely and Strategic Action to Inform ICD-11 Policy. Phase I Findings Report. Accessed February 18, 2025, https://ncvhs.hhs.gov/wp-content/uploads/2023/05/ICD-11-WG-Phase-I-Findings-Report.pdf
- On page 2, the following sentence was revised as follows:
  - Original: “One focus of the US initiative has been understanding how ICD-11 could impact payment models, particularly diagnosis-related groups (DRGs) and other billing and reimbursement processes, which directly influence reimbursement under Medicare and other payers; however, limitations in specificity sometimes result in inaccuracies in risk adjustment.”
  - Revised: “One focus of the US initiative was to explore how ICD-11 could impact payment models, particularly diagnosis-related groups (DRGs) and other billing and authorization processes, which directly influence payment under Medicare and other payers.”
- On page 2, the following sentence was revised as follows:
  - Original: “Transitioning to ICD-11 is expected to enable more precise categorization of patient conditions, thereby refining DRG assignments and leading to more equitable reimbursement that aligns closely with actual patient complexity and resource utilization.”
  - Revised: “Transitioning to ICD-11 is expected to enable more precise categorization of patient conditions, thereby leading to more equitable reimbursement that aligns closely with actual patient complexity and resource utilization.”
- On page 2, the following sentence was revised as follows:
  - Original: “By carefully assessing how ICD-10-CM is being used, and clarifying its strengths and limitations, the Workgroup has sought to identify areas where ICD-11 might address these limitations, such as improving coding specificity, abandoning underused precoordinated codes, and enhancing compatibility with emerging health informatics platforms.”
  - Revised: “By carefully assessing how ICD-10-CM is being used, and clarifying its strengths and limitations, the Workgroup sought to identify areas where ICD-11 might address these limitations, such as improving coding specificity, abandoning underused precoordinated codes, and enhancing compatibility with emerging health informatics platforms.”
    ■ The following citation was also added as reference 11: NCVHS Update Workgroup on Timely and Strategic Action to Inform ICD-11 Policy. 19 September, 2024. Accessed February 18, 2025, https://ncvhs.hhs.gov/wp-content/uploads/2024/10/Presentation-Full-Committee-Meeting-September-20-2024-Ferguson.pdf
- On page 2, the present perfect tense was changed to the past tense in the following sentences:
  - “Under the National Committee on Vital and Health Statistics (NCVHS), the United States established a dedicated ICD-11 Workgroup tasked with assessing the feasibility, regulatory considerations, and resource needs for adoption.”
  - “As a multi-stakeholder body, NCVHS facilitated partnerships across federal agencies, healthcare providers, and industry representatives to tackle the specific challenges that ICD-11 presents for the US health system, especially with regard to existing statutory and regulatory frameworks.”
  - “National Committee on Vital and Health Statistics prioritized timely and well-informed action so that the United States can avoid repeating the costs and burdens associated with its delayed implementation of ICD-10 for morbidity reporting, including the development of a full US-specific clinical modification as occurred for ICD-10.”
- Reference 9 was updated as follows:
  - National Committee on Vital and Health Statistics. Workgroup on timely and strategic action to inform ICD-11 policy. ICD-11 Workgroup Charge. Accessed February 18, 2025, https://web.archive.org/web/20250121184217/https://ncvhs.hhs.gov/subcommittees-work-groups/icd-11-workgroup/
- References 10-14 were renumbered as 12-16 due to the addition of references 10 and 11.

Given the nature of the changes and that no other results or conclusions were impacted by this error, the authors are retracting the original version of the article and replacing it with a new version. The new version of this article has been reviewed and accepted by the editors and has been republished at https://doi.org/10.1093/haschl/qxaf099.

